# Effects of socioeconomic factors and booking time on the WHO recommended eight antenatal care contacts in Liberia

**DOI:** 10.1371/journal.pgph.0000136

**Published:** 2022-02-28

**Authors:** Michael Ekholuenetale, Chimezie Igwegbe Nzoputam, Amadou Barrow

**Affiliations:** 1 Faculty of Public Health, Department of Epidemiology and Medical Statistics, College of Medicine, University of Ibadan, Ibadan, Nigeria; 2 Department of Public Health, Center of Excellence in Reproductive Health Innovation (CERHI), College of Medical Sciences, University of Benin, Benin City, Nigeria; 3 Department of Medical Biochemistry, School of Basic Medical Sciences, University of Benin, Benin City, Nigeria; 4 Department of Public & Environmental Health, School of Medicine & Allied Health Sciences, University of The Gambia, Kanifing, The Gambia; Federal University of Bahia, BRAZIL

## Abstract

Antenatal care (ANC) is an important intervention that has been linked to reduce maternal and newborn adverse outcomes. However, the long years of war in Liberia may have contributed to the poor health indices including the uptake of maternal health care services. The objective of this study was to determine the marginal interaction effects between booking time and socioeconomic factors in eight or more ANC contacts. A total sample of 4,185 women who had given birth were included in this study. The 2020 Liberia Demographic and Health Survey (LDHS) dataset was analyzed. The outcome variable was eight or more ANC contacts. Percentage and Chi-square test were used in univariate and bivariate analyses respectively. The marginal interaction effects between booking time and socioeconomic factors of eight or more ANC contacts were estimated. The statistical significance was determined at 5%. The weighted prevalence of eight or more ANC contacts was 26.6% (95% CI: 23.8%, 29.6%). The uptake of eight or more ANC contacts increased steadily by increasing women’s level of education and household wealth index. Women with higher educational attainment had a prevalence of 49.0% (95%CI: 36.5%, 61.6%) and those in the richest households had an estimated prevalence of 31.4% (95%CI: 24.9%, 38.8%) respectively. Furthermore, the urban dwellers had a weighted eight or more ANC contacts prevalence of 29.0% (95%CI: 24.6%, 34.0%). The key finding is increased marginal interaction effects for higher education and early booking (48.4%), richest households and early booking (35.4%), and urban residential status and early booking (36.2%) respectively. Overall, the prevalence of eight or more ANC contacts was low. However, we found higher coverage of eight or more ANC contacts among women who initiated ANC within the first trimester and among those with higher socioeconomic status. We recommend the Liberian government to design and/or support programmes targeted at promoting early ANC initiation and supporting the disadvantaged women such as the uneducated, poor and those living in rural or remote settings.

## Introduction

Pregnancy-related problems account for more than half of all women deaths in many resource-poor settings every year. World Health Organization (WHO) reported that 9 of every 10 maternal death are recorded in resource-constrained settings [1]. The report of maternal deaths have prompted a global call for immediate action, as the majority of these deaths can be avoided with skilled birth attendance and increased antenatal care (ANC) contacts [2]. In 2016, WHO made a recommendation to increase the frequency of ANC contacts with skilled medical personnel, from four to eight [3–5]. This was seen as a potential strategy to reduce maternal deaths by up to 8 per 1000 live births [6]. This recommendation is believed to aid the implementation of preventive measures, especially early detection of danger signs, reduction of complications, and the elimination of health care inequities, particularly in the rural and marginalized areas [7]. The guideline could increase the chance to improve maternal health services uptake including; dietary counseling, blood test, use of intermittent preventative therapy in pregnancy (IPTp) and the tetanus immunization [6].

The staggering maternal deaths is disproportionately higher among women of low socioeconomic class [1,8,9]. The disadvantaged women and their newborns continue to die due to complications during pregnancy, childbirth or the weeks following delivery (puerperium), especially in developing countries, such as sub-Saharan Africa (SSA) and southern Asia. The global maternal mortality ratio (MMR) declined from 385 deaths per 100,000 livebirths in 1990, to 216 in 2015, corresponding to a relative decline of 43·9%, with 303,000 maternal deaths worldwide [8]. Meanwhile, the regional MMRs was 546 deaths per 100,000 livebirths for SSA [8]. This is worrisome and requires accelerated progress to achieve the Sustainable Development Goals (SDGs) targeted at reducing maternal mortality [10].

It is difficult to estimate the socioeconomic burden due to maternal death especially in resource-constrained settings [11]. Several factors have been identified to be associated with maternal deaths. For example, inadequate access to maternal health care services, increases the risk of pregnancy and obstetric issues. The uptake of adequate ANC contacts is influenced by several factors such as socioeconomic factors [12], maternal age [7], educational level [13], intended pregnancies [14], women’s enlightenment and timing of first ANC visit [2]. In addition, exposure to media, family income, and obstetric service accessibility have been linked to higher prenatal care service uptake [14,15].

There has been a growing interest in the supply and accessibility of maternal health care services in developing countries, particularly those that have recently emerged from long years of civil conflict, such as Liberia. The goal is to undertake concerted efforts to reduce maternal and newborn mortality, which has continued to be a major problem [8], especially that disparities in the distribution of maternal health care services exist in Liberia. For example, about 90% of women in the highest income group had a delivery attended by skilled personnel as compared to 43% of women in the lowest income group [16]. Liberia’s MMR is among the highest in the world. The key attributable factor is inadequate ANC visit [16].

The prolonged civil war that ravaged the country came to an end in 2003. During the conflict, the healthcare system was ravaged, with the majority of healthcare institutions devastated, thereby leaving the country with some of the worst health indices in the world. Due to the massive collapse of healthcare system, women became more reliant on alternative healthcare services, such as traditional healers [17]. The poor healthcare system [18] is a notable effect of the two Liberian civil wars (between 1989 and 1996 and 1997–2003 respectively). After years of civil war and the worst Ebola outbreak in history, the Liberian health system has struggled to rebuild a comprehensive maternal health care.

Liberia is currently ranked as one of the countries with the highest MMR worldwide, with a staggering record of approximately 725 deaths per 100,000 live births [19]. Currently, Liberia is facing supply-side factors such as inadequate infrastructure, lack of competent and sufficient health care providers and medical equipment [20]. However, the health sector is making efforts to revamp the healthcare system and improve women’s access to health care services. Since the end of the civil unrest, the Liberian government has begun a large-scale effort to reconstruct the healthcare system like some other developing countries [21]. By selecting high-priority services, a cost-effective and evidence-based intervention to improve maternal health has been linked to increase access to health care and lower morbidity and mortality rates [22]. To our best of knowledge, no study has been found in Liberia that looked at the coverage and factors associated with the revised WHO ANC guideline of eight ANC contacts. The aim of this study was to investigate the marginal interaction effect between ANC booking time and socioeconomic factors in eight ANC contacts in Liberia.

## Methods

### Ethics statement

We used secondary data available in the public domain. The procedure for the 2019/20 LDHS has been approved by the ICF Institutional Review Board and the Liberia Health Service Ethical Review Committee. Participants have been informed of the benefits and risks of participating in the survey. Written informed consent was obtained directly from qualified respondents prior to administration of the Household or Women’s Questionnaire. The survey was entirely voluntary. The final datasets did not include the respondents’ names or identification numbers. No further approval was required for this study. More details about data and ethical standards are available at http://goo.gl/ny8T6X.

### Data source

The datasets used in this study were obtained from the Liberia Demographic and Health Survey for 2019/2020 (LDHS). The individual women dataset, which included large representative samples of women aged 15–49 years, was used in the analysis. This study examined data from 4185 women. To obtain a nationally representative sample of respondents, a questionnaire for individual women aged 15–49 was used. USAID commissioned the population-based surveys, which were carried out by the Liberian government with operational assistance from ICF International. The survey use a two-stage stratified cluster design. Clusters or Enumeration Areas (EA) were first chosen from a population sample frame, and households were then systematically chosen from the clusters. The MEASURE DHS programme developed standardized questionnaires for households, men, and women, which were administered during face-to-face interviews. The details of DHS data has been report in a previous study [23].

### Variable selection and measurement

#### Outcome

The frequency of ANC contacts with physicians, nurses, and midwives was measured on a dichotomous scale. The LDHS asked the question *"Number of antenatal visits during pregnancy*?" This question’s responses were classified as < 8 or ≥8 contacts. The WHO ANC guideline recommendations map to the eight suggested contacts and provide a review structure for the 2016 WHO ANC [3–5].

#### Socioeconomic variables

Women’s educational attainment, household wealth quintiles, residential and employment status were used to assess socioeconomic status in accordance with previous research [24–26]. Women’s educational attainment was classified as follows: no formal education, primary, secondary, or higher. The location of residence: urban vs. rural. Women’s employment was classified as either employed or unemployed. The wealth indicator weights were assigned using the principal component analysis (PCA) technique. Wealth indicator variable scores were assigned and standardized based on household assets such as wall type, floor type, roof type, water supply, sanitation facilities, radio, electricity, television, refrigerator, cooking fuel, furniture, and room occupancy. The factor loadings and z-scores were then calculated. The indicator values were multiplied by the factor loadings for each household and summarized to produce the household’s wealth index value. The standardized z-score was used to divide the overall scores into wealth quintiles: poorest, poorer, middle, richest, and richest [27].

#### Explanatory variables

The independent variables include: total children ever born: 1–2, 3–4, 5+; health insurance coverage: covered vs. not covered; religion: Christianity, Islam and traditional/others; region: North Western, South Central, South Eastern A, South Eastern B and North Central; marital status: single, currently married/in union, formerly married; age: 15–19, 20–24, 25–29, 30–34, 35–39, 40–44, 45–49; wanted child when became pregnant: then, later, no more; ANC booking time: late (after 1^st^ trimester), early (within 1^st^ trimester).

### Analytical approach

The survey module (’svy’) command was used to modify the sampling design. A variance inflation factor of 10 was used to determine multicollinearity, which is known to be a major source of concern in regression models [28,29]. However, no variable was removed from the model because it was determined that they were not interdependent. Percentage was used in univariate analysis. The Chi-square test was used to investigate the relationship between eight ANC contacts and the explanatory variables. Statistical significance was determined at p< 0.05. Stata version 14 (StataCorp., College Station, TX, USA) was used for data analysis.

### Marginal effect modelling

A marginal effect is the change in the predicted value of eight or more ANC contacts after changing an explanatory factor, either a discrete change in categorical variables or an instantaneous change in continuous variables, while all other variables are held at specified values. The predictive marginal effect model included all significant variables from the bivariate analysis (with corresponding 95% CI). The predictive marginal effect model is presented thus;

$$\mathrm{Pr}(Y=1|\mathrm{Set}[E=e])=\underset{z}{\sum }\;{\hat{p}}_{ez}\;\mathrm{Pr}(Z=z);$$

Where Set[E = e] reflects putting all observations to a single exposure level e, and Z = z refers to a given set of observed values for the covariate vector Z. Furthermore, ${\hat{p}}_{ez}$ is the predicted probabilitis of **≥** 8 ANC contacts for any E = e and Z = z. The marginal effects indicate a weighted average over the distribution of the covariates and are equal to estimates got by standardizing to the entire population. As a post logit test, the exposure *E* is set to the level *e* for all women in the dataset, and the logit coefficients are used to compute predicted probabilities for every woman at their observed covariate pattern and newly exposure value. Because predicted probabilities are computed under the same distribution of *Z*, there is no covariate of the corresponding effect measure estimates [30,31].

## Results

The weighted prevalence of eight or more ANC contacts was 26.6% (95% CI: 23.8%, 29.6%). In Table 1, the uptake of eight or more ANC contacts increased steadily by increasing women’s level of education and household wealth quintile; with women in higher educational attainment having a prevalence of 49.0% (95%CI: 36.5%, 61.6%) and those in the richest households having an estimated prevalence of 31.4% (95%CI: 24.9%, 38.8%) respectively. Furthermore, the urban dwellers had a weighted eight or more ANC contacts prevalence of 29.0% (95%CI: 24.6%, 34.0%). In addition, those who booked early (initiated ANC contacts within 1^st^ trimester) had a prevalence of 34.7% (95%CI: 31.3%, 38.1%). Women who planned for pregnancy and those currently married/in union had the highest eight or more ANC contacts prevalence of 30.7% (95%CI: 27.4%, 34.3%). See Table 1 below for the details.

**Table 1:** Distribution of eight or more ANC contacts in Liberia (n = 4,185).

Variable	n (%)	Prevalence of eight or more ANC contacts	P
**Socioeconomic variables**			
**Education**			<0.001*
No formal education	1688 (40.3)	24.2 (21.5, 27.2)	
Primary	1231 (29.4)	24.4 (21.0, 28.1)	
Secondary	1181 (28.2)	27.6 (23.6, 32.0)	
Higher	85 (2.0)	49.0 (36.5, 61.6)	
**Household wealth quintile**			0.042*
Poorest	1109 (26.5)	24.8 (20.9, 29.1)	
Poorer	914 (21.8)	22.3 (19.1, 25.8)	
Middle	825 (19.7)	24.8 (19.4, 31.0)	
Richer	712 (17.0)	30.7 (25.4, 36.6)	
Richest	625 (14.9)	31.4 (24.9, 38.8)	
**Residential status**			0.033*
Urban	1493 (35.7)	29.0 (24.6, 34.0)	
Rural	2692 (64.3)	23.4 (21.0, 26.0)	
**Employment**			0.233
Employed	2790 (66.7)	24.9 (21.0, 29.2)	
Unemployed	1395 (33.3)	27.5 (24.4, 30.6)	
**Other women’s characteristics**			
**Total children ever born**			0.018*
1–2	1726 (41.2)	26.7 (22.8, 31.0)	
3–4	1092 (26.1)	30.3 (26.3, 34.7)	
5+	1367 (32.7)	22.7 (19.4, 26.3)	
**Health insurance coverage**			0.479
Covered	128 (3.1)	31.4 (18.5, 48.0)	
Not covered	4057 (96.9)	26.5 (23.7, 29.4)	
**Religion**			0.160
Christianity	3556 (85.0)	26.0 (23.0, 29.1)	
Islam	541 (12.9)	31.4 (24.8, 38.9)	
Traditional/others	88 (2.1)	18.2 (8.2, 35.7)	
**Region**			0.530
North Western	626 (15.0)	24.6 (20.3, 30.0)	
South Central	1098 (26.2)	28.1 (22.8, 34.1)	
South Eastern A	630 (15.1)	28.2 (23.1, 33.9)	
South Eastern B	738 (17.6)	26.1 (22.0, 30.7)	
North Central	1093 (26.1)	24.9 (21.6, 28.5)	
**Marital status**			0.034*
Single	888 (21.2)	22.8 (17.7, 28.9)	
Currently married/in union	2910 (69.5)	28.7 (25.8, 31.7)	
Formerly married	387 (9.3)	22.1 (16.8, 28.4)	
**Age**			0.011*
15–19	470 (11.2)	20.2 (15.1, 26.6)	
20–24	971 (23.2)	27.8 (23.5, 32.6)	
25–29	897 (21.4)	26.9 (22.4, 32.0)	
30–34	690 (16.5)	32.4 (27.2, 38.1)	
35–39	665 (15.9)	24.5 (19.5, 30.3)	
40–44	356 (8.5)	25.8 (19.6, 33.1)	
45–49	136 (3.3)	13.2 (7.6, 21.9)	
**Wanted child when became pregnant**			<0.001*
Then	2429 (58.0)	30.7 (27.4, 34.3)	
Later	1384 (33.1)	22.6 (19.1, 26.5)	
No more	372 (8.9)	15.2 (11.1, 20.4)	
**ANC Booking time**			<0.001*
Early (within 1^st^ trimester)	2955 (72.2)	34.7 (31.3, 38.1)	
Late (after 1^st^ trimester)	1140 (27.8)	7.4 (5.0, 10.6)	

*Significant at p<0.05.

In Table 2, we presented the predictive marginal interaction effect between booking time and education, household wealth and residential status in eight or more ANC contacts. The marginal predictive analysis was conducted to decipher the effects of socioeconomic factors with eight or more ANC contacts while adjusting for other maternal characteristics. From the predictive marginal effects results, assuming that the distribution of all factors remained the same, but every woman had higher education, we would expect 42.3% of eight or more ANC contacts. If every woman booked early (within 1^st^ trimester), we would expect 34.2% of eight or more ANC contacts. If instead, every women had higher education and booked late (after 1^st^ trimester), we would expect 26.3% of eight or more ANC contacts. Moreover, if every woman had higher education and booked early (within 1^st^ trimester), we would expect 48.4% of eight or more ANC contacts. By implication, we found increased marginal interaction effect between higher education and early booking than higher education and late booking. If instead, the distribution of other factors remained the same among women, but every woman is in the richest household and booked early (within 1^st^ trimester), we would expect 35.4% of eight or more ANC contacts. If every woman is in the richest household and booked late (after 1^st^ trimester), we would expect about 8.9% of eight or more ANC contacts. Furthermore, if instead, the spread of the aforementioned variables was as observed while all covariates remain equal, but every woman lived in the urban residence and booked early (within 1^st^ trimester), we would expect 36.2% of eight or more ANC contacts, but if all woman lived in the urban residence and booked late, we would expect 8.6% of eight or more ANC contacts. The key finding is increased marginal interaction effects for higher education and early booking (48.4%), richest households and early booking (35.4%), and urban residential status and early booking (36.2%) respectively. The details of the predictive marginal interaction effects of the factors associated with eight or more ANC are shown in Table 2 below.

**Table 2:** Marginal interaction effect of socioeconomic factors and eight or more ANC contacts in Liberia.

Variable	Marginal effect	95% Confidence Interval	P
**Socioeconomic variables**			
**Education**			
No formal education	25.3	21.7, 28.8	<0.001*
Primary	25.7	21.6, 29.8	<0.001*
Secondary	27.6	24.2, 31.0	<0.001*
Higher	42.3	30.0, 54.6	<0.001*
**Booking time**			
Early (within 1^st^ trimester)	34.2	30.9, 37.5	<0.001*
Late (after 1^st^ trimester)	8.0	5.0, 11.0	<0.001*
**Education-booking time interaction**			
No formal education*late booking	5.9	2.0, 9.8	0.003*
No formal education*early booking	32.5	28.2, 36.9	<0.001*
Primary*late booking	8.9	2.1, 11.7	0.005*
Primary*early booking	32.8	27.6, 38.0	<0.001*
Secondary*late booking	8.1	4.0, 12.1	<0.001*
Secondary*early booking	34.9	30.6, 39.2	<0.001*
Higher*late booking	26.3	1.4, 51.2	<0.001*
Higher*early booking	48.4	34.6, 62.7	<0.001*
**Household wealth quintile**			
Poorest	27.5	23.2, 31.7	<0.001*
Poorer	23.8	20.6, 26.9	<0.001*
Middle	26.3	20.7, 31.9	<0.001*
Richer	29.5	24.3, 34.7	<0.001*
Richest	28.2	21.8, 34.5	<0.001*
**Household wealth quintile-booking time interaction**			
Poorest*late booking	7.9	3.3, 12.5	0.001*
Poorest*early booking	34.9	29.5, 40.3	<0.001*
Poorer*late booking	4.9	1.6, 8.1	0.003*
Poorer*early booking	30.8	26.5, 35.1	<0.001*
Middle*late booking	7.2	1.9, 12.4	0.007*
Middle*early booking	33.5	26.0, 40.9	<0.001*
Richer*late booking	10.9	4.2, 17.6	0.001*
Richer*early booking	36.5	30.5, 42.6	<0.001*
Richest*late booking	8.9	-0.01, 18.6	0.076
Richest*early booking	35.4	28.6, 42.3	<0.001*
**Residential status**			
Urban	28.6	24.1, 33.1	<0.001*
Rural	24.9	22.3, 27.6	<0.001*
**Residential status-booking time interaction**			
Urban*late booking	8.6	4.3, 12.8	<0.001*
Urban*early booking	36.2	30.7, 41.7	<0.001*
Rural*late booking	7.2	2.9, 11.6	0.001*
Rural*early booking	31.6	28.4, 34.8	<0.001*
**Other women’s characteristics**			
**Total children ever born**			
1–2	26.6	22.8, 30.4	<0.001*
3–4	28.5	24.5, 32.5	<0.001*
5+	26.2	20.7, 31.7	<0.001*
**Marital status**			
Single	23.1	18.2, 27.9	<0.001*
Currently married/in union	29.0	25.9, 32.1	<0.001*
Formerly married	24.4	18.4, 30.4	<0.001*
**Age**			
15–19	26.9	18.9, 34.9	<0.001*
20–24	29.5	24.5, 34.4	<0.001*
25–29	25.7	21.4, 30.1	<0.001*
30–34	29.0	24.1, 33.9	<0.001*
35–39	24.2	18.8, 29.6	<0.001*
40–44	28.4	21.3, 35.6	<0.001*
45–49	16.1	8.1, 24.2	<0.001*
**Wanted child when became pregnant**			
Then	29.3	26.1, 32.5	<0.001*
Later	24.6	20.7, 28.5	<0.001*
No more	19.8	14.2, 25.4	<0.001*

*Significant at p<0.05.

**Fig 1:**
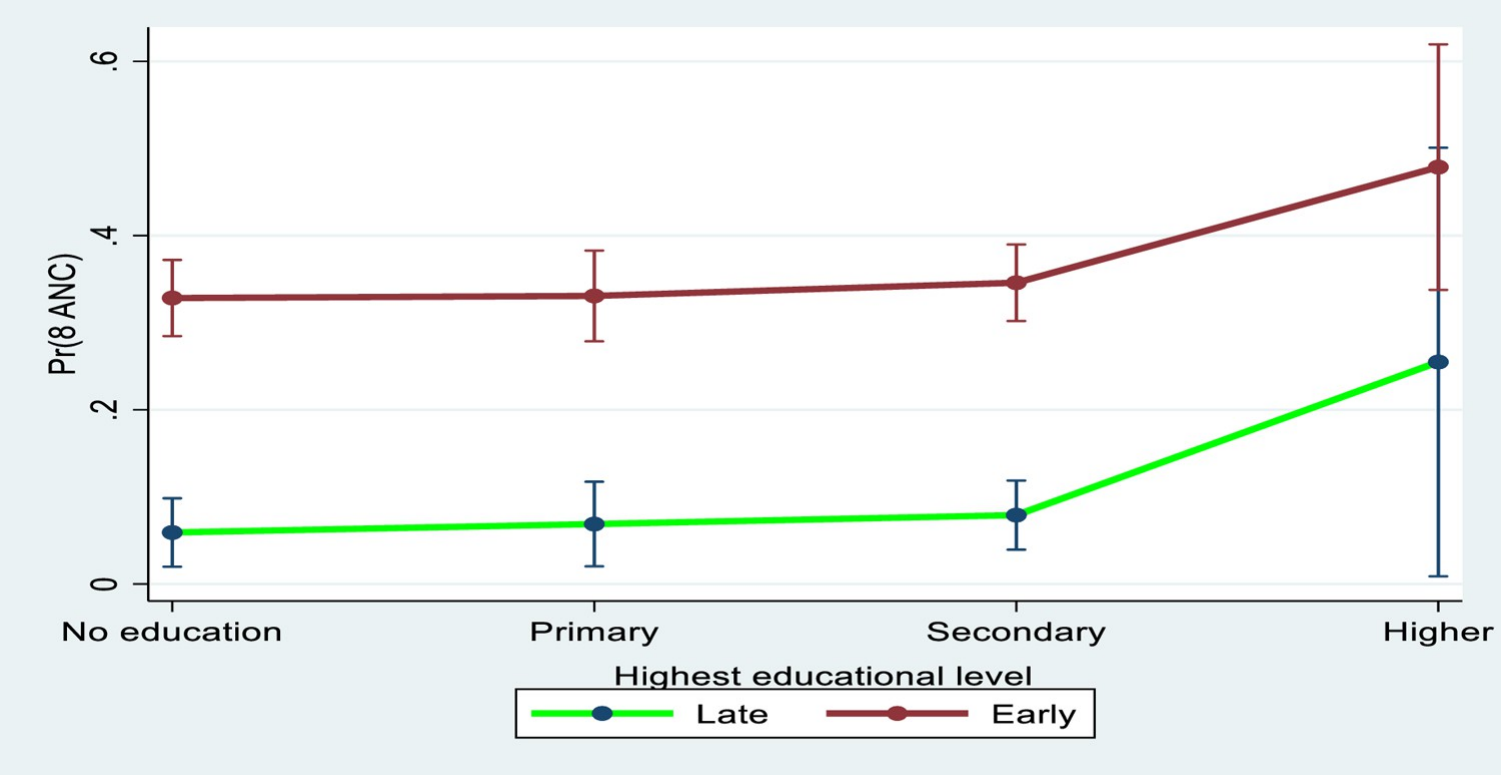
Predictive marginal effects between early booking and educational attainment in eight or more ANC contacts.

Fig 1 showed the marginal effects plot between booking time and educational attainment in eight or more ANC contacts. The marginal interaction effects in eight or more ANC contacts among the Liberian women was higher for women who booked early than those who booked late. Notably, the marginal interaction effects in eight or more ANC contacts was higher among women who booked early (within 1^st^ trimester) across various educational levels (higher, secondary, primary and without formal education).

**Fig 2:**
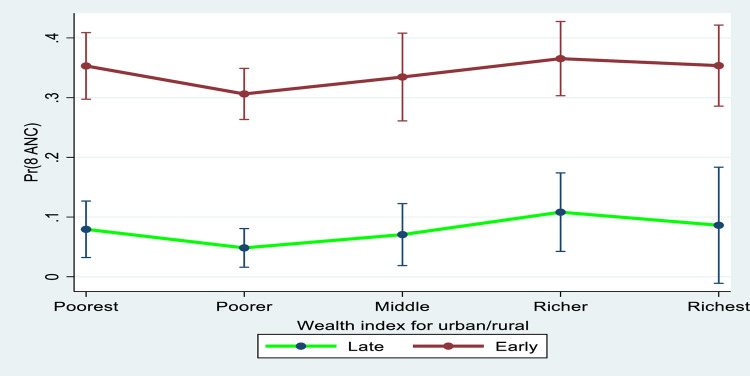
Predictive marginal effects between early booking and household wealth quintile in eight or more ANC contacts.

In Fig 2, we presented the marginal effects plot between booking time and household wealth in eight or more ANC contacts. The marginal interaction effects in eight or more ANC contacts, was higher for women who booked early than those who booked late. In addition, the marginal interaction effects in eight or more ANC contacts was higher among women who booked early (within 1^st^ trimester) across household wealth quintiles (poorest, poorer, middle, richer and richest).

**Fig 3:**
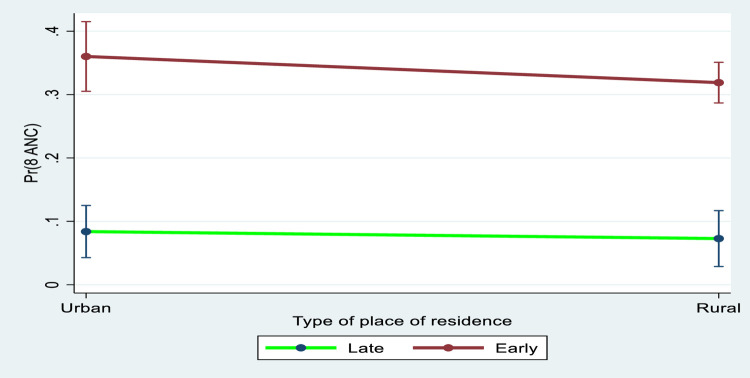
Predictive marginal effects between early booking and residential status in eight or more ANC contacts.

Fig 3 revealed the marginal effects plot between booking time and residential status in eight or more ANC contacts. The marginal interaction effects in eight or more ANC contacts was higher for women who booked early than those who booked late. Furthermore, the marginal interaction effects in eight or more ANC contacts was higher among women who booked early (within 1^st^ trimester) in urban and rural residence.

## Discussion

This is the foremost study to assess the recent WHO ANC guideline using the 2020 LDHS. Approximately one-quarters of women had eight or more ANC contacts. This prevalence is grossly inadequate considering the maternal mortality burden in Liberia. The prevalence of eight or more ANC contacts in this study is higher than the previous report in some other countries, such as Senegal (1.0%), Uganda (1.0%), Zambia (1.0%), Mozambique (2.0%), Mali (3.0%), Guinea (3.0%), Cameroon (8.0%), Benin (9.0%), Papua New Guinea (10.0%), Pakistan (14.0%), and Nigeria (20.0%) [32]. Few other countries, such as Albania (30.0%), Ghana (44.0%), and Jordan (74.0%) showed a greater prevalence of eight or more ANC contacts [32]. The low prevalence reported from the previous multi-country study was expected in our study given that the majority of the study countries were low- and middle-income, where health care services are commonly found to be inadequate, substandard or non-existent.

Also, the economic development index of Liberia is similar to that of other resource-constrained settings. The availability and uptake of mother and child health care services in low- and middle-income countries are inadequate due to a lack of infrastructure, poor levels of education, low enlightenment, poverty, even when services are accessible, women are forced to pay out-of-pocket which hinders many in the uptake. To improve maternal health care service utilization, improving the infrastructure, policy formation in the areas of maternal and child health, women empowerments and comprehensive health insurance coverage, particularly for reproductive-age women are required.

More specifically, our research found that higher education and early antenatal bookings had a greater marginal effect in eight or more ANC contacts. When compared with women with lower educational attainment who booked early, those with higher educational attainment and who booked early had greater eight or more ANC uptake. This means that educational attainment and early ANC booking have a positive interaction effect. From the least educated to the most educated, there was a considerable positive interaction effect with early booking. The interaction effects clearly demonstrate that scheduling ANC within the first trimester, will enhance women’s uptake of eight or more ANC contacts. The implication is that early ANC booking and improvement in maternal education will help to achieve the WHO’s recommended eight or more ANC contacts. From previous studies [33–35], education and enlightenment are major positive factors that enhance women’s uptake of maternal and child care services. Other studies have also validated the assertion that women with higher educational attainment are more likely to use maternal health care services, when compared with their counterparts [36–39]. To improve the level of knowledge or enlightenment, girl-child education should be given utmost importance. Women should be exposed to educational materials and other awareness campaign channels such as watching television and listening to health information on radio amongst other behaviour change communication media. No doubt, exposure to health information will contribute to women’s knowledge of the importance of early booking and completion of eight or more ANC contacts for a successful pregnancy.

The interaction between household wealth and booking time in eight or more ANC contacts was also investigated. The results showed increased marginal interaction effects between the wealthiest households and early bookings in eight or more ANC contacts. By implication, women who come from the most affluent households and book early will have a greater uptake of ANC contacts. The uptake of ANC and other maternal care services has been positively impacted by wealth status especially in countries such as Liberia where less than 5% of the women are reportedly covered by health insurance. According to a recent study, women from wealthy families are more likely to access and use maternal health care services, when compared with women from low income families [40]. Moreover, women from wealthy households are able to afford health care services especially in out-of-pocket health expenses situation [41]. Besides intervention to promote early booking, improving women’s wealth status should also be a priority. To address the issues surrounding poverty in low uptake of health care services, the concept of Universal Health Coverage (UHC) was launched to promote health and well-being and to extend life expectancy for all through access to quality health care [42]. This assumes that no one is to be left behind. Achieving UHC entails providing everyone with complete access to high-quality healthcare services, as well as financial risk protection.

Promoting health insurance coverage in Liberia will be a mechanism for increasing ANC contacts. At the time of ANC service, there should be no deductibles, copayments, coinsurance payments, or other additional payments. Out-of-pocket expenses could be a significant impediment to making at least eight ANC contacts. A study comparing health care expenditures across SSA found that the majority of countries reported catastrophic health expenditures, with out-of-pocket expenses ranging from one-fifth to more than two-thirds of total health expenditures, indicating that health care remains largely inaccessible despite the introduction of health insurance schemes [43]. This could also be the situation.

Also, we found a significant positive marginal interaction effect between urban residence and early bookings in eight or more ANC contacts. Women who lived in the urban area and booked early had higher coverage for eight or more ANC contacts, when compared with those who lived in rural area. This suggests a link between living in urban residence and booking early in eight or more ANC contacts. Previous studies also found urban dwellers had more ANC contacts than their rural counterparts [44,45]. This urban-rural inequalities in maternal health care service uptake is a major concern. This finding is common in many resource-constrained settings due to disparity in the distribution of functional health facilities which is usually in favour of the urban residence. In addition to promoting early booking, health facilities in rural, remote and hard-to-reach neighbourhoods should be revamped with infrastructure, health care personnel and comprehensive services as commonly available in the urban residence.

To improve optimal uptake of ANC contacts, it is important to design and implement comprehensive maternal health programs and policies. The illiterate, poor and women who live in hard-to-reach communities should be targeted for improved ANC service use. The provision of maternal health care services and health insurance coverage have been recommended [44,46]. Out-of-pocket payments for maternal health services will remain a major barrier to health care access [33], except the Liberian government rise up to this challenge. Based on the findings of this study, if a woman lacks formal education, poor, or lives in a rural residence, early booking alone will not be sufficient to obtain the eight or more ANC contacts.

### Strength and limitation

A significant strength of this study was the use of nationally representative high-quality data from a household survey. Furthermore, appropriate statistical adjustments for the survey designs ensure that the results of this study are reliable. Notwithstanding, a significant limitation of this paper is the possibility of recall bias, which could result in an over- or under-estimation of eight or more ANC contacts. LDHS also does not collect household income or expenditure data, which are the traditional metrics for calculating wealth. The asset-based wealth index used here is merely a proxy indicator of household economic status, and it does not always produce results that are comparable to those obtained from direct income and expenditure measurements when such data is available or can be collected accurately.

## Conclusion

There was low uptake of eight or more ANC contacts in Liberia, as only about one-quarters of women had met the recommended frequency of ANC contacts. We found higher coverage of eight ANC visits among women who had early ANC initiation (within the first trimester) and among those with higher socioeconomic status. We recommend the Liberian government to design and/or support programmes targeted at promoting early ANC initiation (i.e within first trimester) and supporting the disadvantaged women such as the uneducated, poor and those living in rural or remote settings.

## Abbreviations

ANC: Antenatal Care
CI: Confidence Interval
EAs: Enumeration Areas
LDHS: Liberia Demographic and Health Survey
ICF: Inner City Fund
IPTp: Intermittent Preventative Therapy in Pregnancy
MMR: Maternal Mortality Ratio
PCA: Principal Components Analysis
SDGs: Sustainable Development Goals
SE: Standard Error
TX: Texas
UHC: Universal Health Coverage
USA: United States of America
WHO: World Health Organization

## References

[1] World Health Organization. Trends in maternal mortality 2000 to 2017: estimates by WHO, UNICEF, UNFPA, World Bank Group and the United Nations Population Division. Geneva: World Health Organization; 2019. Available: https://apps.who.int/iris/handle/10665/327595.

[2] Ekholuenetale M, Nzoputam CI, Barrow A, Onikan A. Women’s enlightenment and early antenatal care initiation are determining factors for the use of eight or more antenatal visits in Benin: further analysis of the Demographic and Health Survey. J Egypt Public Health Assoc. 2020;95: 13. doi: 10.1186/s42506-020-00041-2 32813174 PMC7364685

[3] Tunçalp Ӧ., Pena-Rosas J, Lawrie T, Bucagu M, Oladapo O, Portela A, et al. WHO recommendations on antenatal care for a positive pregnancy experience-going beyond survival. BJOG Int J Obstet Gynaecol. 2017;124: 860–862. doi: 10.1111/1471-0528.14599 28190290

[4] de Masi S, Bucagu M, Tunçalp Ö, Peña-Rosas JP, Lawrie T, Oladapo OT, et al. Integrated Person-Centered Health Care for All Women During Pregnancy: Implementing World Health Organization Recommendations on Antenatal Care for a Positive Pregnancy Experience. Glob Health Sci Pract. 2017;5: 197–201. doi: 10.9745/GHSP-D-17-00141 28655799 PMC5487083

[5] World Health Organization, editor. WHO recommendations on antenatal care for a positive pregnancy experience. Geneva: World Health Organization; 2016. 28079998

[6] WHO Reproductive Health Library. WHO recommendation on antenatal care contact schedules. WHO Reprod Health Libr Geneva World Health Organ. 2018; 7.

[7] Rahman A, Nisha MK, Begum T, Ahmed S, Alam N, Anwar I. Trends, determinants and inequities of 4+ ANC utilisation in Bangladesh. J Health Popul Nutr. 2017;36: 2. doi: 10.1186/s41043-016-0078-5 28086970 PMC5237328

[8] Alkema L, Chou D, Hogan D, Zhang S, Moller A-B, Gemmill A, et al. Global, regional, and national levels and trends in maternal mortality between 1990 and 2015, with scenario-based projections to 2030: a systematic analysis by the UN Maternal Mortality Estimation Inter-Agency Group. The Lancet. 2016;387: 462–474. doi: 10.1016/S0140-6736(15)00838-7 26584737 PMC5515236

[9] Chol C, Negin J, Agho KE, Cumming RG. Women’s autonomy and utilisation of maternal healthcare services in 31 Sub-Saharan African countries: results from the demographic and health surveys, 2010–2016. BMJ Open. 2019;9: e023128. doi: 10.1136/bmjopen-2018-023128 30867200 PMC6429737

[10] Rosa W, editor. Transforming Our World: The 2030 Agenda for Sustainable Development. A New Era in Global Health. New York, NY: Springer Publishing Company; 2017. doi: 10.1891/9780826190123.ap02

[11] Lucas A, Pons C. Maternal and Reproductive Health: An Epidemic of Inequality. In: ISGlobal [ 12 Jun 2017 [cited 25 Sep 2021]. Available: https://www.isglobal.org/en/epidemia-desigualdad.

[12] Ekholuenetale M, Nzoputam CI, Barrow A. Prevalence and Socioeconomic Inequalities in Eight or More Antenatal Care Contacts in Ghana: Findings from 2019 Population-Based Data. Int J Womens Health. 2021;Volume 13: 349–360. doi: 10.2147/IJWH.S306302 33790659 PMC8001171

[13] Ali N, Sultana M, Sheikh N, Akram R, Mahumud RA, Asaduzzaman M, et al. Predictors of Optimal Antenatal Care Service Utilization Among Adolescents and Adult Women in Bangladesh. Health Serv Res Manag Epidemiol. 2018;5: 2333392818781729. doi: 10.1177/2333392818781729 30083573 PMC6069020

[14] Tekelab T, Chojenta C, Smith R, Loxton D. Factors affecting utilization of antenatal care in Ethiopia: A systematic review and meta-analysis. Lassi ZS, editor. PLOS ONE. 2019;14: e0214848. doi: 10.1371/journal.pone.0214848 30973889 PMC6459485

[15] Djoukou Olga Denise K, Mélèdje Marie-Dorothée K, Yacé Marie-Laurette A, Akoua T-K, Esme Marie Laure E, Williams Y, et al. Factors Associated with Maternal Health Service Utilization in Cote d’Ivoire: Analysis of the 2011 Ivorian Demographic and Health Survey. Sci J Public Health. 2019;7: 115. doi: 10.11648/j.sjph.20190704.12

[16] Landau AS, Bender JA, Waters AE, Ulku AS, Ly EJ. Tackling maternal mortality in rural Liberia: a field and facility-based approach. Ann Glob Health. 2016;82: 475. doi: 10.1016/j.aogh.2016.04.301

[17] Johnson K, Asher J, Rosborough S, Raja A, Panjabi R, Beadling C, et al. Association of combatant status and sexual violence with health and mental health outcomes in postconflict Liberia. JAMA. 2008;300: 676–690. doi: 10.1001/jama.300.6.676 18698066

[18] Svoronos T, Macauley RJ, Kruk ME. Can the health system deliver? Determinants of rural Liberians’ confidence in health care. Health Policy Plan. 2015;30: 823–829. doi: 10.1093/heapol/czu065 25070742

[19] WHO WHO. Global Health Observatory, Liberia Health System. 2016 [cited 25 Sep 2021]. Available: https://www.who.int/data/gho/data/countries/country-details/GHO/liberia?countryProfileId=cda05b49-7f7f-424f-bb80-4125392e9424.

[20] Kentoffio K, Kraemer JD, Griffiths T, Kenny A, Panjabi R, Sechler GA, et al. Charting health system reconstruction in post-war Liberia: a comparison of rural vs. remote healthcare utilization. BMC Health Serv Res. 2016;16: 478. doi: 10.1186/s12913-016-1709-7 27604708 PMC5015243

[21] Loevinsohn B, Harding A. Buying results? Contracting for health service delivery in developing countries. Lancet Lond Engl. 2005;366: 676–681. doi: 10.1016/S0140-6736(05)67140-1 16112305

[22] Roberts B, Guy S, Sondorp E, Lee-Jones L. A basic package of health services for post-conflict countries: implications for sexual and reproductive health services. Reprod Health Matters. 2008;16: 57–64. doi: 10.1016/S0968-8080(08)31347-0 18513607

[23] Corsi DJ, Neuman M, Finlay JE, Subramanian S. Demographic and health surveys: a profile. Int J Epidemiol. 2012;41: 1602–1613. doi: 10.1093/ije/dys184 23148108

[24] Szklarska A, Jankowska EA. Independent effects of social position and parity on body mass index among Polish adult women. J Biosoc Sci. 2003;35: 575–583. doi: 10.1017/s002193200300600x 14621253

[25] Walters S, Suhrcke M. Socioeconomic inequalities in health and health care access in central and eastern Europe and the CIS. 2005; 50.

[26] Bado AR, Sathiya Susuman A. Women’s Education and Health Inequalities in Under-Five Mortality in Selected Sub-Saharan African Countries, 1990–2015. Carpenter DO, editor. PLOS ONE. 2016;11: e0159186. doi: 10.1371/journal.pone.0159186 27442118 PMC4956109

[27] Rutstein SO, Staveteig S. Making the Demographic and Health Surveys Wealth Index Comparable. 2014; DHS Methodological Reports No. 9. Rockville, Maryland, USA: ICF International.

[28] Midi H, Sarkar SK, Rana S. Collinearity diagnostics of binary logistic regression model. J Interdiscip Math. 2010;13: 253–267. doi: 10.1080/09720502.2010.10700699

[29] Fox J, Monette G. Generalized Collinearity Diagnostics. J Am Stat Assoc. 1992;87: 178–183. doi: 10.1080/01621459.1992.10475190

[30] Williams R. Using the Margins Command to Estimate and Interpret Adjusted Predictions and Marginal Effects. Stata J Promot Commun Stat Stata. 2012;12: 308–331. doi: 10.1177/1536867X1201200209

[31] Ekholuenetale M, Adeyoju TO, Onuoha H, Barrow A. Renal Failure among Women of Reproductive Age in Burundi: Estimating the Prevalence and Associated Factors Using Population-Based Data. Kershaw DB, editor. Int J Nephrol. 2021;2021: 1–11. doi: 10.1155/2021/6640495 33728065 PMC7936884

[32] Ekholuenetale M. Prevalence of Eight or More Antenatal Care Contacts: Findings From Multi-Country Nationally Representative Data. Glob Pediatr Health. 2021;8: 2333794X211045822. doi: 10.1177/2333794X211045822 34527769 PMC8436290

[33] Meda IB, Baguiya A, Ridde V, Ouédraogo HG, Dumont A, Kouanda S. Out-of-pocket payments in the context of a free maternal health care policy in Burkina Faso: a national cross-sectional survey. Health Econ Rev. 2019;9: 11. doi: 10.1186/s13561-019-0228-8 30919219 PMC6734235

[34] Kalu-Umeh NN, Sambo MN, Idris SH, Kurfi AM. Costs and Patterns of Financing Maternal Health Care Services in Rural Communities in Northern Nigeria: Evidence for Designing National Fee Exemption Policy. Int J MCH AIDS. 2013;2: 163–172. doi: 10.21106/ijma.21 27621969 PMC4948141

[35] Perkins M, Brazier E, Themmen E, Bassane B, Diallo D, Mutunga A, et al. Out-of-pocket costs for facility-based maternity care in three African countries. Health Policy Plan. 2009;24: 289–300. doi: 10.1093/heapol/czp013 19346273 PMC2699243

[36] Shibre G, Mekonnen W. Socio-economic inequalities in ANC attendance among mothers who gave birth in the past 12 months in Debre Brehan town and surrounding rural areas, North East Ethiopia: a community-based survey. Reprod Health. 2019;16: 99. doi: 10.1186/s12978-019-0768-8 31286965 PMC6615209

[37] Ekholuenetale M, Benebo FO, Idebolo AF. Individual-, household-, and community-level factors associated with eight or more antenatal care contacts in Nigeria: Evidence from Demographic and Health Survey. PLOS ONE. 2020;15: e0239855. doi: 10.1371/journal.pone.0239855 32976494 PMC7518602

[38] Novignon J, Ofori B, Tabiri KG, Pulok MH. Socioeconomic inequalities in maternal health care utilization in Ghana. Int J Equity Health. 2019;18: 141. doi: 10.1186/s12939-019-1043-x 31488160 PMC6729067

[39] Nwosu CO, Ataguba JE. Socioeconomic inequalities in maternal health service utilisation: a case of antenatal care in Nigeria using a decomposition approach. BMC Public Health. 2019;19: 1493. doi: 10.1186/s12889-019-7840-8 31703734 PMC6842188

[40] Tunçalp Ӧ., Pena-Rosas J, Lawrie T, Bucagu M, Oladapo O, Portela A, et al. WHO recommendations on antenatal care for a positive pregnancy experience-going beyond survival. BJOG Int J Obstet Gynaecol. 2017;124: 860–862. doi: 10.1111/1471-0528.14599 28190290

[41] Ekholuenetale M, Barrow A. Inequalities in out-of-pocket health expenditure among women of reproductive age: after-effects of national health insurance scheme initiation in Ghana. J Egypt Public Health Assoc. 2021;96: 6. doi: 10.1186/s42506-020-00064-9 33704601 PMC7952482

[42] Kieny MP, Bekedam H, Dovlo D, Fitzgerald J, Habicht J, Harrison G, et al. Strengthening health systems for universal health coverage and sustainable development. Bull World Health Organ. 2017;95: 537–539. doi: 10.2471/BLT.16.187476 28670019 PMC5487973

[43] World Health Organization. Global health observatory data repository: Health expenditures ratios by country, 1995–2014. 2014 [cited 1 Oct 2019]. Available: http://apps.who.int/nha/database.

[44] Dahiru T, Oche OM. Determinants of antenatal care, institutional delivery and postnatal care services utilization in Nigeria. Pan Afr Med J. 2015;21. doi: 10.11604/pamj.2015.21.321.6527 26587168 PMC4633744

[45] Laaser U, Broniatowski R, Byepu S, Bjegovic-Mikanovic V. Delays in Achieving Maternal, Newborn, and Child Health Targets for 2021 and 2030 in Liberia. Front Public Health. 2019;7: 386. doi: 10.3389/fpubh.2019.00386 31921750 PMC6923216

[46] Dahab R, Sakellariou D. Barriers to Accessing Maternal Care in Low Income Countries in Africa: A Systematic Review. Int J Environ Res Public Health. 2020;17: 4292. doi: 10.3390/ijerph17124292 32560132 PMC7344902

