# Supplementary material for: Effects of socioeconomic factors and booking time on the WHO recommended eight antenatal care contacts in Liberia
Source: PLOS Glob Public Health. 2022 Feb 28;2(2):e0000136. doi: 10.1371/journal.pgph.0000136 (PMC10022028; doi:10.1371/journal.pgph.0000136)
Supplement: Attachment — Submitted filename: Response to Reviewers.docx [file pgph.0000136.s001.docx]

December 4, 2021

**Manuscript Reference#: PGPH-D-21-00801**

**Title:** “Effects of socioeconomic factors and booking time on the WHO recommended eight antenatal care contacts in Liberia”

### RE: Revised manuscript submission and response to reviewer’s comments

Dear Editor,

This letter is in reference to your email dated December 4, 2021 with reviewer’s comments. We are very pleased that the manuscript is potentially acceptable for publication in PLOS Global Public Health once we have carried out the revisions.

We would like to thank the reviewer for these insightful and helpful comments and for giving us the chance to revise our manuscript. We believe the revised manuscript has been significantly improved and the reviewer’s comments have been addressed adequately. We think in its current form it will make a valuable contribution to the literature on this increasingly important topic. 

Please find for your kind consideration the followings:

1) A section-by-section response to the comments and suggestions of the reviewers (below).
2) The revised manuscript, provided as a marked-up copy and a clean copy.

We hope that these changes meet with your favourable consideration. Please do not hesitate to get in touch if you require any further information. 

Amadou Barrow 
Corresponding Author 

**Response to reviewer reports:**

Reviewer #1: This study aimed to investigate the marginal interaction effect between ANC booking time and socioeconomic factors in eight ANC contacts in Liberia. The introduction delimits the theme well, exploring the changes in the WHO recommendations for the reduction of maternal deaths. Finally, it highlights the high maternal mortality rates in Sub-Saharan Africa, South Asia and Liberia. This is a study with secondary data from the Liberia Demographic and Health Survey for 2019/2020 (LDHS) that used the number of prenatal visits during pregnancy (<8 or ≥8 contacts) as an outcome. Wealth indicator weights were assigned using the principal component analysis (PCA) technique. Independent variables include: total number of children born; health insurance; religion; region; marital status; age; she wanted a child when she got pregnant; ANC booking time. The Chi-square test was used to investigate the relationship between eight ANC contacts and the Explanatory variables.

**Response**: Thank you very much. We respectfully appreciate your articulated comments.

The predictive marginal effect model included all significant variables from the bivariate analysis (with the corresponding 95% CI). However, the choice of the predictive marginal effect model is not well described in the Methods, especially knowing that the use of these models is more suitable for binary variables with responses (0.1).

**Response**: Thank you very much for the insightful comment. We have included the marginal effect modelling separately in the methods section. We hope this is helpful.

***“Marginal effect modelling***

*A marginal effect is the change in the predicted value of eight or more ANC contacts after changing an explanatory factor, either a discrete change in categorical variables or an instantaneous change in continuous variables, while all other variables are held at specified values. The predictive marginal effect model included all significant variables from the bivariate analysis (with corresponding 95% CI). The predictive marginal effect model is presented thus;*

$\Pr(Y=1\text{|Set[E=e])=}\sum_{\text{z}} \mathop{p}\limits^{\wedge} _{ez}\Pr(Z=z)$*;*

*Where Set[E = e] reflects putting all observations to a single exposure level e, and Z = z refers to a given set of observed values for the covariate vector Z. Furthermore,*$\mathop{p}\limits^{\wedge} _{ez}$*is the predicted probabilities of* ***≥*** *8 ANC contacts for any E = e and Z = z. The marginal effects indicate a weighted average over the distribution of the covariates and are equal to estimates got by standardizing to the entire population. As a post logit test, the exposure E is set to the level e for all women in the dataset, and the logit coefficients are used to compute predicted probabilities for every woman at their observed covariate pattern and newly exposure value. Because predicted probabilities are computed under the same distribution of Z, there is no covariate of the corresponding effect measure estimates”.*

The sample is large enough to estimate marginal effects for standardization for the entire population, and the results presented in Table 2 are revealing. The graphs highlight the differences between education, family wealth and housing. As a suggestion, authors can remove the colored lines, because line graph is more suited to visualizing trends and movements over time numerically and evenly distributed.

**Response**: Thank you very much for the insightful comment. Regarding the removal of coloured lines, and using line graph to depict time series trends visualization, we like to say the graph are NOT line graphs, but interaction plots to decipher the association between the “socioeconomic factors” and “eight ANC” by “timing to ANC initiation/booking (late versus early)”. It is easy for a layman to see that the coverage of eight ANC is higher in early ANC initiation/booking (**brown line**) and among women with higher education and urban settlements. Since the graph was generated using Stata software, and giving the reasons that they are not line graphs, we hope the reviewer would support that we keep the graphs for plausible comparisons/explanations for the interaction effect.

Critical points for review: The choice of the predictive marginal effect model is not well described in the Method section. The results, in part, were discussed with the scientific literature and some limitations pointed out. The conclusion does not primarily respond to the objective of the study, and must be rewritten, taking advantage of the findings in table 2. I do not recommend the publication of the manuscript in its current format, but the study itself shows sufficient potential for authors to be encouraged to resubmit a revised version.

**Response**: Thank you very much for the insightful comment. We have provided the predictive effect modelling section in the methods section. We have now revised conclusion to basically respond to the objective of the study;

*“There was low uptake of eight or more ANC contacts in Liberia, as only about one-quarters of women had met the recommended frequency of ANC contacts. We found higher coverage of eight ANC visits among women who had early ANC initiation (within the first trimester) and among those with higher socioeconomic status. We recommend the Liberian government to design and/or support programmes targeted at promoting early ANC initiation (within first trimester) and supporting the disadvantaged women such as the uneducated, poor and those living in rural or remote settings”.*
